# Effects of N^6^-Methyladenosine Modification on Cancer Progression: Molecular Mechanisms and Cancer Therapy

**DOI:** 10.3389/fonc.2022.897895

**Published:** 2022-05-30

**Authors:** Yong-fu Zhu, Shu-Jie Wang, Jie Zhou, Ye-han Sun, You-mou Chen, Jia Ma, Xing-xing Huo, Hang Song

**Affiliations:** ^1^The First Department of Oncology, The First Affiliated Hospital of Anhui University of Chinese Medicine, Hefei, China; ^2^The Department of Acupuncture, The Third Affiliated Hospital of Zhejiang Chinese Medical University, Hangzhou, China; ^3^Anhui Province Key Laboratory of Medical Physics and Technology, Institute of Health and Medical Technology, Hefei Institutes of Physical Science, Chinese Academy of Sciences, Hefei, China; ^4^Experimental Center of Clinical Research, Scientific Research Department, The First Affiliated Hospital of Anhui University of Chinese Medicine, Hefei, China; ^5^Department of Biochemistry and Molecular Biology, School of Integrated Chinese and Western Medicine, Anhui University of Chinese Medicine, Hefei, China

**Keywords:** RNA modifications, N6-methyladenosine, female malignancies, molecular mechanisms, immunotherapy

## Abstract

N^6^-methyladenosine (m^6^A) is a major internal epigenetic modification in eukaryotic mRNA, which is dynamic and reversible. m^6^A is regulated by methylases (“writers”) and demethylases (“erasers”) and is recognized and processed by m^6^A-binding proteins (“readers”), which further regulate RNA transport, localization, translation, and degradation. It plays a role in promoting or suppressing tumors and has the potential to become a therapeutic target for malignant tumors. In this review, we focus on the mutual regulation of m^6^A and coding and non-coding RNAs and introduce the molecular mechanism of m^6^A methylation involved in regulation and its role in cancer treatment by taking common female malignant tumors as an example.

## Introduction

N^6^-methyladenosine (m^6^A) alteration is a methylation modification located on the 6th nitrogen atom of adenine. m^6^A is the most abundant form of epigenetic modification in eukaryotic RNA, which exists in several different types of RNA, including mRNA and non-coding RNA. At present, the research on m^6^A methylation and malignant tumors mainly focuses on the influence of m^6^A methylation on tumor cell proliferation, invasion, resistance to radiotherapy and chemotherapy, and prognosis of patients (1). The detection rate of malignant tumors is growing year by year, and the age of onset tends to be younger, thanks to the popularization of associated cancer screening tools and an increase in people’s health awareness (2). The primary clinical treatment for malignant tumors is to select individualized surgery combined with postoperative radiotherapy and chemotherapy according to the patient’s condition (3). However, for some patients with relapsed and refractory tumors, the treatment effect is often difficult to achieve the expected (4). Therefore, one of the most pressing issues to be addressed is elucidating the etiology of malignant tumors and finding novel therapeutic medications to overcome tumor resistance. In this review, we focus on the effect of m^6^A methylation on the occurrence and development of malignant tumors and introduce the molecular mechanisms involved in the regulation of m^6^A methylation and its role in cancer treatment by taking common female malignancies as an example.

## Overview Of M^6^a Methylation

**Figure 1:**
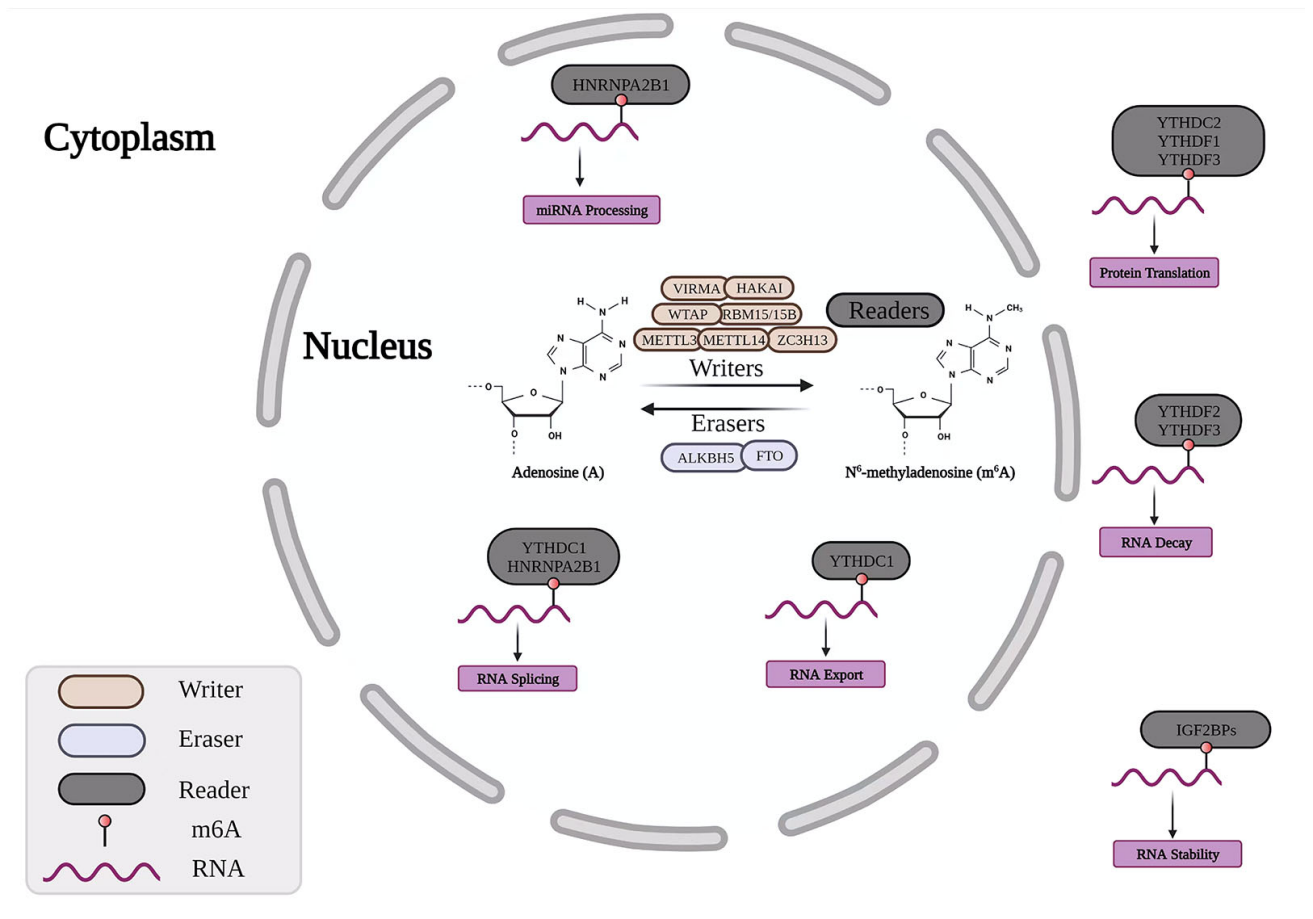
Dynamic m6A RNA modifying process. The RNA modification of m6A is regulated by methyltransferases (“writers”), demethylases (“erasers”) and m6A-binding proteins (“readers”). VIRMA, Vir Like m6A Methyltransferase Associated; HAKAI, Cbl Proto-Oncogene Like 1; WTAP, Wilms Tumor 1 Associated Protein; RBM15, RNA Binding Motif Protein 15; METTL3, Methyltransferase Like 3; METTL14, Methyltransferase Like 14; ZC3H13, Zinc Finger CCCH-Type Containing 13; ALKBH5, AlkB Homolog 5; FTO, Fat Mass And Obesity-Associated Protein; HNRNP, Heterogeneous Nuclear Ribonucleoprotein; YTHDF, YTH domain protein family; IGF2BP, Insulin Like Growth Factor 2 MRNA Binding Protein.

The m^6^A methylation modification of RNA is the most common internal modification in RNA modification. m^6^A is the methylation modification of RNA on the 6th nitrogen atom of adenosine, and the process is dynamic and reversible. The proteins involved in the methylation and demethylation of m^6^A are divided into three categories, namely methyltransferases (“writers”), demethylases (“erasers”), and m^6^A recognition proteins (“readers”) (**Figure 1**). Through RNA transcription, splicing, processing, translation, and degradation, it plays a role in the formation and spread of numerous malignant cancers. (5). Furthermore, studies have shown that m^6^A methylation modification is closely related to the activation and inhibition of cancer-related signaling pathways, which mainly affect tumor progression by regulating related tumor biological functions (6). At present, m^6^A RNA modification is increasingly used in cancer detection and related targeted molecules (7, 8).

### m^6^A Readers

For m^6^A-modified mRNA to perform specific biological functions, a specific RNA-binding protein, methylation reader protein, is required. It mainly includes YTH m^6^A RNA-binding protein (YTH) domain proteins (including YTHDF1, YTHDF2, YTHDF3, YTHDC1, and YTHDC2) (9), heterogeneous nuclear ribonucleoproteins (HNRNPC, HNRNPG, and HNRNPA2B1) and eukaryotic initiation factor (eIF). The functions of these reader proteins are mainly to alter protein-RNA interactions by impairing the homologous binding of m^6^A to RNA-binding proteins and altering RNA secondary structure (10). Studies have found that YTHDF1 has a clear oncogenic role, and its high expression in cancer genes can accelerate the transformation of important oncogenic drivers in cancer *via* numerous methods, impacting cancer progression and prognosis. For example, in gastric cancer progression, mutated YTHDF1 enhances the expression of the key oncogenic factor Wnt receptor Frizzled7 (FZD7), leading to gastric cancer progression and poor prognosis (11).

### m^6^A Writers

The m^6^A methyltransferase consists of methyltransferase-like 3 (METTL3), METTL14, WTAP, RBM15, ZC3H13, VIRMA, and the newly discovered METTL16, which are also called writers. Its primary function is to catalyze the m^6^A modification of adenylate on mRNA. METTL3 and METTL14 have a critical catalytic domain (12), and the METTL3-METTL14 methyltransferase complex assembly during m^6^A modification is a crucial factor initiating the modification. In addition, methyltransferases also include associated protein (WTAP), RBM15, ZC3H13, vir-like m^6^A methyltransferase associated (VIRMA), and METTL16. These methyltransferases all play an important role in forming METTL3-METTL14 complexes in different links, affecting cancer cell proliferation and migration (13–16).

### m^6^A Erasers

m^6^A modification is the earliest reversible modification found among many RNA modifications, and the reversibility of its modification is due to the existence of demethylases. In addition, their encoding genes are called “erasers”. Fat mass and obesity-associated protein (FTO) is the first demethylase discovered and is a member of the Alkb protein family, which can affect the RNA-binding ability of the splicing factor serine and arginine-rich splicing factor 2 (SRSF2), thereby regulating the splicing process of pre-mRNA (17). ALKBH5, another member of the Alkb protein family, was found to have a demethylation function, which can directly catalyze m^6^A-methylated adenosine to remove methyl groups, different from the oxidative demethylation of FTO (18).

## The Role Of M^6^a Modifications In Non-Coding Rnas

In recent years, m^6^A has been found to exist in various ncRNAs such as miRNAs, long non-coding RNAs (lncRNAs), circular RNAs (circRNAs), ribosomal RNAs (rRNAs), and small nuclear RNAs (snRNAs), essential for its metabolism and function (19). Moreover, some m^6^A regulatory proteins associated with aberrant m^6^A modification of ncRNAs are also involved in cancer cells proliferation, invasion, and drug resistance, suggesting a potential link between cancer and m^6^A-ncRNA modification (20).

### The Effect of m^6^A on LncRNA

LncRNAs, a type of ncRNAs of 200 or more nucleotides, can regulate gene expression at multiple levels (21). Many m^6^A-modified lncRNAs have been found recently, and they can control gene expression and function in a variety of ways. They can act as transcriptional regulators, acting in cis or trans, regulating the transcription of adjacent genes (22). XIST, one of the first functionally annotated lncRNAs, plays a key role in X chromosome inactivation by recruiting multiple factors (23). A study shows that in head and neck squamous cell carcinoma, METTL3- and METTL14-mediated m^6^A methylation contributes to the stability of LNCAROD, and LNCAROD overexpression promotes the malignant development of HNSCC by promoting YBX1-hspa1a interaction, thereby enhancing the stability of the YBX1 protein (24). Another study found that modifying lncRNAs with m^6^A had the opposite effect on cancers. METTL14 suppressed colorectal cancer growth and metastasis by downregulating oncogenic long non-coding RNA XIST in the METTL14-YTHDF2-lncRNA regulatory axis (25). LncRNA GAS5 binds directly to Yes-associated protein (YAP), promoting its phosphorylation and ubiquitin-mediated degradation, thereby attenuating YAP-mediated transcription of YTHDF3 and inhibiting the progression of rectal cancer (26). LincRNA-p21 acts as a tumor inhibitor in the development of esophageal squamous cell carcinoma (27). In hepatocellular carcinoma, the m^6^A methylation modification of LINC00152 is involved in the prognosis of LIHC patients through the cytoskeleton regulation pathway (28). Furthermore, in rectal cancer, m^6^A modification of lncRNA RP11 can upregulate the translation of Zeb1 to trigger cancer cell dissemination (29); lncRNA-THOR enhances IGF2BP1-targeted mRNA expression and promotes human osteosarcoma cell survival and proliferation (30).

### The Effect of m^6^A Modification on MiRNA

MiRNAs are non-coding single-stranded RNAs of 21-25 nucleotides in length that regulate gene expression at the post-transcriptional level by building RNA-induced silencing complexes (RISCs) that bind to the 3’untranslated region of target mRNAs (3’UTR) to regulate gene expression (31). In the nucleus, miRNAs are first transcribed into longer primary miRNAs (pri-miRNAs) and subsequently processed into precursor miRNAs (pre-miRNAs). It is then cleaved into mature single-stranded miRNAs by Dicer in the cytoplasm, and the participation and processing of such pri-miRNAs are m^6^A-dependent. METTL3 tags pre-miRNAs through m^6^A modification, enabling DGCR8 to recognize and bind its specific substrates, thereby promoting miRNA maturation and increased miRNA levels in cells (32).

### The Role of m^6^A Modification of CircRNA in Cancer

Circular RNAs (circRNAs) are a class of single-stranded covalently closed RNA molecules that participate in many physiological processes, including competing with endogenous RNAs as sponge miRNAs, forming RNA-protein complexes, regulating gene transcription, and even encoding proteins (33). In most cases, abnormal m^6^A modification contributes to tumorigenesis and tumor progression. However, m^6^A modification on circRNAs can suppress innate immunity; YTHDF2 sequesters m^6^A-circRNA and is essential for suppressing innate immunity (34). Chen et al. found that m^6^A modification of circNSUN2 promoted liver metastasis of colorectal cancer by promoting cytoplasmic export and forming a circNSUN2/IGF2BP2/HMGA2 RNA-protein triple complex to stabilize HMGA2 mRNA (35).

## The Role Of M^6^a Modifications In Coding RNAS

m^6^A affects all physiological processes such as mRNA processing, nuclear export, translation, and degradation. It mainly affects mRNA stability, which is also closely related to the occurrence and development of malignant tumors. At present, the research on m^6^A and its participants in the reversible regulation process (m^6^A-modifying enzymes and m^6^A-binding proteins) and the mechanism of tumorigenesis and development has gradually become a hot spot.

Ries et al. found that m^6^A-mRNA is regulated by compartments, including mRNA stability and reduced translation. This study demonstrates that the number and distribution of m^6^A sites in cellular mRNA can modulate and influence the composition of the phase-separated transcriptome (36). Li et al. first elucidated the *in vivo* biological role of m^6^A modification in T cell-mediated pathogenesis. They revealed a novel mechanism for T cell homeostasis and signal-dependent induction of mRNA degradation (37). RNA methyltransferase (METTL3) acts as a translation initiation complex, thereby enhancing the translation of target mRNAs (38). In addition, at different intracellular locations, m^6^A exerts methyltransferase activity-dependent and -independent functions in gene regulation. Besides, the RNA methyltransferase METTL16 is in the nucleus, acting as an m^6^A writer, depositing m^6^A into its hundreds of a specific messenger RNA target. In the cytosol, METTL16 promotes translation in an m^6^A-independent manner (39).

## The Role Of M^6^a Modifications In Common Female Malignancies

Common malignant tumors in women mainly include breast cancer (BC), ovarian cancer (OC), cervical cancer (CC), and endometrial cancer (EC). Despite advancements in examination methods in the prevention and treatment of common female malignant tumors, most patients are in the middle and late stages of their disease due to difficulties in early diagnosis and localization of tumors and a lack of effective efficacy evaluation and prognosis monitoring methods. Therefore, the mortality rate of common malignant tumors in women continues to increase. Genetic, epigenetic, and environmental factors drive its occurrence, development, and transfer, and epigenetic factors play an important role as a bridge between genetic and environmental factors. Epigenetics has multiple forms of expression, of which m^6^A is the most abundant form of internal modification. In this review, we take common female malignant tumors as examples to introduce the molecular mechanism of m^6^A modification in the occurrence and development of cancer and its application in cancer treatment (**Table 1**).

**Table 1:** Dysregulation of m6A modification in common female malignant (CFM).

m^6^A regulators	Target	Regulation in CFM	Fuction	Mechanisms
METTL3	RAGE	Down	writer	METTL3 increases cisplatin chemosensitivity of cervical cancer cells *via* downregulation of the activity of RAGE. Li, R. et al. (40);
	MALAT1	Down	writer	The m6A methyltransferase METTL3 controls epithelial-mesenchymal transition, migration and invasion of breast cancer through the MALAT1/miR-26b/HMGA2 axis. Zhao, C et al. (41)
WTAP	HBS1L/FAM76A	Up	writer	Identification of WTAP-related genes by weighted gene co-expression network analysis in ovarian cancer. Wang, J. et al. (42).
YTHDF1	RANBP2	Up	reader	YTHDF1 Aggravates the Progression of Cervical Cancer Through m(6)A-Mediated Up-Regulation of RANBP2. Wang, H. et al. (43).
	EIF3C	Up	reader	The m^6^A reader YTHDF1 promotes ovarian cancer progression *via* augmenting EIF3C translation. Liu, T. et al. (9).
eIF3	WNT	Up	reader	The Immune-Related Gene ELF3 is a Novel Biomarker for the Prognosis of Ovarian Cancer. Xu, H., et al. (44).
FTO	miR-181b-3p	Up	ereaser	The FTO/miR-181b-3p/ARL5B signaling pathway regulates cell migration and invasion in breast cancer. Xu, Y. et al. (45).
	BNIP3	Up	ereaser	RNA N6-methyladenosine demethylase FTO promotes breast tumor progression through inhibiting BNIP3. Niu, Y. et al. (46).
	cAMP	Down	ereaser	FTO-Dependent N (6)-Methyladenosine Modifications Inhibit Ovarian Cancer Stem Cell Self-Renewal by Blocking cAMP Signaling. Huang, H. et al. (47).
ALKBH5	NANOG	Up	ereaser	RNA demethylase ALKBH5 promotes ovarian carcinogenesis in a simulated tumour microenvironment through stimulating NF-kappaB pathway. Jiang, Y. et al. (48).

### The Regulatory Role of m^6^A Methyltransferase (Writers)

m^6^A methyltransferases (“Writers”) are an essential class of catalytic enzymes. METTL3 is a key regulator that promotes m^6^A modification, and the abnormal regulation of METTL3 is also inextricably linked to tumor development. Some studies have confirmed that the overexpression of METTL3 may be an important factor in promoting the development of common malignant tumors in women. Pan et al. found that the expression of RBM15 and METTL3 in CESC (cervical squamous cell carcinoma) tissues was higher than that in normal tissues (49). In addition, Hua et al. found that METTL3 promoted the epithelial-to-mesenchymal transition of ovarian cancer cells and the proliferation, invasion, and tumor formation of ovarian cancer cells, affecting their prognosis and overall survival (50). Moreover, Ma et al. compared the expressions of METTL14, WTAP, and METTL3 in ovarian cancer and found that METTL3 independently regulates m^6^A modification and thus affects the proliferation and metastasis (51). Besides, Li et al. found that METTL3 inhibited the viability of cervical cancer cells and enhanced their sensitivity to the chemotherapeutic drug cisplatin by downregulating the expression of the receptor for advanced glycation and its product in cervical cancer tissues (40); METTL3 modulated the m^6^A modification of MALAT1. The expression of MALAT1 is upregulated, and MALAT1 can promote the expression of high mobility group A2(HMGA2) by sponge miR-26b, thereby promoting the development of breast cancer (41). Regarding this, Wang et al. proposed that WTAP may promote the proliferation, invasion, and migration of ovarian cancer through two gene sequences of FAM76A and HBS1 (42).

### The Regulatory Role of m^6^A Methylation Reader Proteins (Readers)

As m^6^A methylation reading proteins, “Readers” can recognize the information of RNA methylation modification and participate in downstream mRNA translation, degradation, and miRNA processing. It mainly changes the interaction between protein and RNA by weakening the homologous binding of m^6^A to RNA-binding protein and changing the secondary structure of RNA.

YTHDF1 acts as an important reading element in m^6^A modification by recognizing m^6^A-containing mRNAs and promoting their translation initiation and elongation (52). Wang et al. applied online data analysis to identify RANBP2 as a critical target of YTHDF1 in cervical cancer cells, and subsequent reduction of RANBP2 decreased cervical cancer cell proliferation, migration, and invasion (43). Overexpression of YTHDF1 promoted the growth, migration, and invasion of Hela and Siha cells. At the same time, knockdown of RANBP2 reversed the effect of overexpression of YTHDF1 on cervical cancer progression, indicating that YTHDF1 promotes cervical cancer progression by regulating RANBP2 expression in an m^6^A-dependent manner. Some scholars have proposed that YTHDF1 directly targets eIF3C (a subunit of EIF3) and promotes ovarian cancer’s occurrence, metastasis, and prognosis (51).

Heterogeneous ribonucleoproteins (hnRNPs) are a diverse family of RNA-binding proteins that function in most stages of RNA metabolism (53). Studies have shown that HNRNPC regulates target transcripts’ abundance and alternative splicing through mA binding to RNA. Other researchers have proposed that hnRNPA2B1 can inhibit the growth of ovarian cancer cells, reduce the mobility of ovarian cancer cells *in vitro*, and hinder the formation of xenograft tumors *in vivo*. In addition, hnRNPA2B1 promotes the occurrence and development of malignant phenotypes of ovarian cancer by activating the expression of Lin28B (54). Moreover, Shi et al. found that lobaplatin induced apoptosis and cell cycle arrest by downregulating hnRNP A2/B1 in cervical cancer cells, and knockdown of hnRNP A2/B1 significantly reduced tumor growth in nude mice xenografts and increased cervical cancer Cellular sensitivity to lobaplatin and irinotecan (55). Eukaryotic initiation factor 3 (eIF3) can bind to m^6^A-modified bases in the 5’ UTR of RNA, promoting mRNA translation (56). The high expression of eLF3 in ovarian cancer is closely related to its poor prognosis (44); Zhu et al. concluded that eIF3B is highly expressed in cervical cancer tissues and is closely related to advanced FIGO in cervical cancer patients staging, shorter overall survival and lymph node metastasis (57).

### The Regulatory Role of m^6^A Demethylases (erasers)

Demethylase is an integral part of the reversible modification of m^6^A, and FTO, as the first discovered demethylase, is widely present in adult and embryonic tissues, and its expression is exceptionally high in the brain. Moreover, recent studies have shown that FTO has an important effect on glioblastoma growth and self-renewal (58).

The expression level of FTO is also elevated in cervical squamous cell carcinoma, which can enhance chemoradiotherapy resistance *in vitro* and *in vivo* by reducing m^6^A-regulated β-catenin expression (59). Zhao et al. believed that FTO accelerated the growth of cancer cells by promoting proliferation, inhibiting apoptosis, and activating autophagy in ovarian cancer (60). In the latest study, Huang et al. found that FTO expression in high-grade serous ovarian cancer (HGSOC) tumor cells were significantly lower than that in other tissues, and it had a significant inhibitory effect on ovarian cancer cells (47). It can be seen that FTO may have a bidirectional regulatory impact on ovarian cancer tissue, and the specific mechanism needs to be further studied. Furthermore some researchers believe that FTO primarily stimulates the oncogenic activity of breast cancer cell invasion and migration through the FTO/miR-181b-3p/ARL5B signaling pathway, promoting tumor proliferation (61). The tumor suppressor BNIP3 is a downstream target of FTO-mediated m^6^A modification. FTO mediates m^6^A demethylation in the 3’UTR of BNIP3 mRNA and induces its degradation through a YTHDF2-independent mechanism, promoting breast cancer cell proliferation, colony formation, and *in vitro* and *in vivo* transfer (46).

Among them, ALKBH5, another member of the Alkb protein family, was found to have demethylation, which can directly catalyze m^6^A-methylated adenosine to remove methyl groups is different from the oxidative demethylation of FTO (62). ALKBH5 affects tumor growth by regulating cell proliferation, migration, invasion, and metastasis. Recently, some scholars have proposed that ALKBH5 has dual roles in various cancers. ALKBH5 can reverse METTL3 autophagy in cells through down-regulation of mRNA stability. In epithelial ovarian cancer cells (63), overexpressed ALKBH5 in human ovarian cancer (SKOV3) cells enhanced the stability of BCL-2 mRNA and inhibited tumor cell growth and metastasis. In contrast, overexpressed ALKBH5 in A2780 cells had the exact opposite effect. In cervical cancer tissues, GAS5-AS1 was also low-expressed and inhibited the proliferation and metastasis of cervical cancer cells. And regulate GAS5 expression by interacting with RNA demethylase ALKBH5, thereby inhibiting CC cell proliferation, migration, and invasion (64).

## M^6^A Rna Modifications And Common Female Malignancies Therapy

### The Role of m^6^A RNA Modification Targeted Drugs in the Treatment of Common Female Malignant Tumors

Targeted therapy is at the cellular and molecular level to design corresponding therapeutic drugs for the already defined carcinogenic sites. The drug enters the body and will specifically select the carcinogenic sites to combine and act so that the tumor cells specifically die instead of normal tissue cells surrounding the tumor are affected. Early studies of targeting strategies based on m^6^A modulators have focused on demethylases. Besides, previous studies have shown that m^6^A plays an essential role in the occurrence and development of tumors. Therefore, it is of great scientific significance and clinical value to develop specific inhibitors of m^6^A-related proteins. As the first discovered RNA-modifying demethylase, FTO is widely involved in various physiological processes, and its dysregulation is associated with multiple human diseases.

Due to its involvement in obesity and obesity-induced metabolic diseases and the occurrence, development and prognosis of various cancers, such as melanoma, acute myeloid leukemia, glioblastoma, lung cancer, hepatocellular carcinoma and breast cancer, studies have shown that rhein can induce apoptosis (65). Huang et al.. systematically investigated the effect of rhein on adipogenesis by transcriptional and post-transcriptional approaches and found that rhein regulates m^6^A methylation rearrangement and adipogenesis in an independent manner, inhibiting fat mass and obesity-related (FTO) demethylase activity (66). It is indicated that rhein can inhibit the demethylation activity of FTO on m^6^A on mRNA *in vitro* and *in vivo*, thereby increasing the level of m^6^A in cells. ALKBH5 and FTO are both m^6^A demethylases. Studies have found that in ovarian cancer, the core cytokine NANOG is a key target to promote the development of ovarian cancer (48). On the contrary, there is much evidence to prove the overexpression of METTL3 in tumor tissues (67), while studies targeting METTL3 have shown that it can effectively inhibit tumor growth, proliferation, and metastasis (68).

In the progression of common malignant tumors in women, drug resistance that often occurs in the later stage is also a significant difficulty in its treatment. Chemotherapy resistance, especially platinum resistance, is a major cause of poor prognosis in ovarian cancer. Bowen Li et al. found that m^6^A can modulate the modification of anticancer drug resistance by modulating drug-target interactions and drug-mediated cell death signaling (69). The ethyl ester form of the FTO inhibitor Meclofenaic (MA2) inhibits FTO and enhances the effect of the chemotherapeutic drug temozolom by targeting the MYC-miR-155/23a cluster-MXI1 feedback circuit in gliomas anti-tumor effects (45) (**Table 2**).

**Table 2:** Therapeutic targets of M6A modification in common female malignant (CFM).

Remedy	Regulation of target	Target	Mechanisms
Rhein	inhibition	REEP3	FTO regulates the chemo-radiotherapy resistance of cervical squamous cell carcinoma (CSCC) by targeting beta-catenin through mRNA demethylation. Zhou, S. et al. (59).
Temozolomide	inhibition	MYC-miR-155/23a	FTO Inhibition Enhances the Antitumor Effect of Temozolomide by Targeting MYC-miR-155/23a Cluster-MXI1 Feedback Circuit in Glioma. Xiao, L. et al. (45).
Immune checkpoint inhibitor	inhibition	PD-L1, PD-L2, TIM3, and CCR4	Expression pattern of m(6)A regulators is significantly correlated with malignancy and antitumor immune response of breast cancer. He, X., et al. (70).
Immune checkpoint inhibitor PARP	upregulating	Wnt/β-catenin pathway	N(6)-Methylation of Adenosine of FZD10 mRNA Contributes to PARP Inhibitor Resistance. Fukumoto, T. et al. (71).

### m^6^A RNA Modification and Immunotherapy

The tumor microenvironment (TME) is primarily responsible for mediating immunotherapy responses in tumor progression, and bioinformatics research has shown that m^6^A alteration and its regulators may regulate the TME and are linked to immune checkpoint inhibition (ICB) (7, 72).

Yi et al. systematically studied head and neck squamous cell carcinoma (HNSCC) compared with adjacent normal pairs, concluded that m^6^A regulators were upregulated in HNSCC, and found that m^6^A regulators were associated with PDL in the tumor immune microenvironment (TIME) (73). The expression of -1 was positively correlated, which may provide a promising target for improving the responsiveness of HNSCC to immunotherapy. In addition, He et al. systematically analyzed RNA-sequencing data of 24 major m^6^A methylation regulators in 775 breast cancer patients from the TCGA database and classified them for overall survival in the lower RNA methylation status group (RM1). The higher methylation status (RM2) group was significantly reduced (70). Moreover, the RM2 group displayed higher expression and higher numbers of tumor-infiltrating CD8^+^ T cells, helper T cells, and activated NK cells. The expressions of PD-L2, TIM3, and CCR4 were lower than those of the RM1 group, so it can be considered that the regulator of m^6^A is closely related to the malignant degree, prognosis, and anti-tumor immune response of breast cancer and can be used as a potential target and biological target for breast cancer immunotherapy.

In addition, anti-PD-1 immunotherapy is effective initially, but its efficacy is significantly reduced later due to FTO-mediated resistance (71). However, recent studies have shown that FTO knockdown can increase tumor sensitivity to anti-PD-1 immunotherapy, thereby improving efficacy (69). Therefore, the combined use of ICB and FTO inhibitors may block the development of drug resistance in individuals who develop adaptive immunity.

## Conclusions

With the rapid development of high-throughput sequencing technology and bioinformatics, m^6^A has been gradually revealed as an important epigenetic modification with reversible properties, modification-related enzyme system, and role in different disease processes. It provides infinite possibilities for subsequent tumor diagnosis and treatment. These m^6^A-modified molecules are expected to become effective early diagnosis and prognostic markers for tumors and potential therapeutic targets, providing new ideas for tumor diagnosis and treatment.

Since m^6^A research provides a new understanding of the molecular mechanisms of tumorigenesis, metastasis, immune response, and drug resistance and promotes the development of new therapeutics, the process from theory to clinical translation still needs to be explored. Currently, the understanding of how m^6^A modification affects immune phenotype is still in its infancy. Although some methylase inhibitors have been discovered so far and provide new targets for tumor drugs, their mechanisms of action *in vitro* and *in vivo* are not fully understood and lack specificity. Therefore, the development of more inhibitors against m^6^A-related proteins brings a new dawn for guiding tumor-targeted therapy based on RNA epigenetics. Targeted intervention in m^6^A modification can promote basic research in related fields, show excellent application prospects in tumor treatment and other disease-related fields, and show important scientific significance in life sciences and new drug discovery.

## Author Contributions

HS and X-xH conceived and designed the study. Y-fZ, S-jW, JZ, Y-hS, JM, and Y-mC collected data and aided in writing the manuscript. HS and Y-fZ edited the manuscript. All authors read and approved the final manuscript.

## Funding

This study was supported by the National Natural Science Foundation of China (No. 81802103, 81803938), Project of High-Level Talents in AHUTCM (Project code: 2019rcZD001), Excellent Young Scholars Project of Natural Science Foundation of Anhui Province in China (grant No. 2108085Y29), Natural Science Research Project of Colleges and Universities in Anhui Province (No. KJ2021A0557), Opening Project of Zhejiang Provincial Preponderant and Characteristic Subject of Key University (Chinese Traditional Medicine), and Zhejiang Chinese Medical University (No.ZYXZD2019004).

## Conflict of Interest

The authors declare that the research was conducted in the absence of any commercial or financial relationships that could be construed as a potential conflict of interest.

## Publisher’s Note

All claims expressed in this article are solely those of the authors and do not necessarily represent those of their affiliated organizations, or those of the publisher, the editors and the reviewers. Any product that may be evaluated in this article, or claim that may be made by its manufacturer, is not guaranteed or endorsed by the publisher.

## Abbreviations

3’ -UTR, 3’-untranslation regions; 5’-UTR, 5’-untranslated region; YTH, YTH m6A RNA-binding protein; ALKBH5, AlkB homolog 5; ALKBH3, AlkB homolog 3; FZD7, Wnt receptor frizzled7; WTAP, associated protein; VIRMA, vir-like m6A methyltransferase associated; CC, Cervical cancer; circRNAs, Circular RNAs; EC, Endometrial cancer; eIF3, Eukaryotic initiation factor 3; FTO, Fat mass and obesity-associated protein; SRSF2, Serine and arginine-rich splicing factor 2; HNSCCs, Head and neck squamous cell carcinoma; HNRNPC, HNRNPG and HNRNPA2B1, Heterogeneous nuclear ribonucleoproteins; HGSOC, High-grade serous ovarian cancer; ICB, Immune checkpoint blockade; YAP, Yes-associated protein; IGFBP7, Insulin-like growth factor-binding protein-7; lncRNAs, Long non-coding RNAs; MA, Meclofenaic; mRNA, Messenger RNA; METTL3, Methyltransferase-like 3; METTL14, Methyltransferase-like 14; SKOV3, human ovarian cancer; m6A, N6-Methyladenosine; NPC, Nasopharyngeal carcinoma; ncRNAs, Non-coding RNAs; OC, Ovarian cancer; HMGA2, high mobility group A2; pre-miRNAs, Precursor miRNAs; pri-miRNAs, Primary miRNAs; PD-1, Programmed cell death protein-1; RBM15, RNA-binding motif protein 15; RBM15B, RNA-binding motif protein 15B; RISCs, RNA-induced silencing complexes; rRNAs, Ribosomal RNAs; snRNAs, Small nuclear RNAs; snoRNAs, Small nucleolar RNAs; TME, Tumor microenvironment; TIME, Tumor immune microenvironment; YTHDC1, YTHDC2, YTHDF1, YTHDF2, and YTHDF3, YTH domain protein family.
